# The coefficient of variation of CT values for predicting lymph node metastasis in extrahepatic cholangiocarcinoma

**DOI:** 10.1371/journal.pone.0357578

**Published:** 2026-09-11

**Authors:** Naokazu Chiba, Yuta Abe, Hirotaka Kojima, Ryota Suda, Masashi Nakagawa, Shigeto Ochiai, Takahiro Gunji, Toru Sano, Satoshi Tabuchi, Tetsuo Ishizaki, Shigeyuki Kawachi

**Affiliations:** 1 Department of Digestive and Transplantation Surgery, Tokyo Medical University Hachioji Medical Center, Tokyo, Japan; 2 Department of Surgery, Keio University School of Medicine, Tokyo, Japan; Fondazione Policlinico Universitario Agostino Gemelli IRCCS, ITALY

## Abstract

Preoperative prediction of lymph node metastasis (LNM) in extrahepatic cholangiocarcinoma (ECC) is crucial for surgical strategies and prognosis, as current imaging methods are insufficient. A total of 149 ECC patients who underwent curative resection were retrospectively analyzed. We measured the Coefficient of Variation (CV) of CT values, ADC of MRI, and SUVmax of PET/CT from multiple lymph nodes. Diagnostic performance for LNM prediction was evaluated using ROC curve analysis. The CV of CT values was the most powerful independent predictor of LNM. Its AUC (0.829) was superior to MRI ADC (0.666) and PET/CT SUVmax (0.701). The CV of CT values showed a sensitivity of 72.8%, specificity of 72.9%, and accuracy of 72.9%. In conclusion, The CV of CT values is a promising and effective parameter for preoperatively predicting LNM in ECC. This can potentially improve patient selection for neoadjuvant therapies and surgical planning.

## Introduction

Extrahepatic cholangiocarcinoma (ECC) is a heterogeneous group of malignant biliary epithelial tumors consisting of common bile duct cancer and hilar cholangiocarcinoma. Cholangiocarcinoma is a relatively rare malignancy, though its global incidence is reportedly increasing. Estimates for biliary tract cancers generally range from less than 0.2 to over 2.24 cases per 100,000 person-years, with higher prevalence observed in Asian populations [1]. Surgical resection is the only potentially curative treatment for non-metastatic ECC. Despite surgical advances, the 5-year overall survival (OS) rate for resected cases typically ranges from 11% to 35% [2]. Large cohort studies have indicated that approximately 26–27% of patients with eCCA present with lymph node metastasis at the time of diagnosis. [3,4]. Consequently, accurate preoperative assessment of LNM is crucial for effective surgical planning and improving patient outcomes. Nonetheless, precise preoperative prediction of LNM poses a substantial challenge.

Various non-invasive imaging techniques such as computed tomography (CT), magnetic resonance imaging (MRI), and positron emission tomography (PET)/CT are routinely used to diagnose and stage cholangiocarcinoma. Previous studies have explored the use of radiomics based on these modalities to predict LNM. For instance, CT-based radiomics models have shown promise in predicting LNM in intrahepatic cholangiocarcinoma [5,6]. Similarly, MRI-based radiomics models, which often incorporate apparent diffusion coefficient (ADC) values from diffusion-weighted imaging (DWI), have been developed to predict LNM in both perihilar cholangiocarcinoma and ECC [3,4]. Furthermore, the utility of PET/CT with standardized uptake value (SUV) in diagnosing and staging hilar cholangiocarcinoma and ECC has been investigated [7,8]. Although PET/CT has demonstrated high specificity in detecting regional LNM, its sensitivity is lower than that of multidetector CT or MRI [8]. Despite these advances, the diagnostic performance of current methods for predicting LNM remains insufficient.

The ability to accurately predict LNM preoperatively offers a substantial clinical advantage. A more precise assessment may facilitate the determination of suitable candidates for neoadjuvant chemotherapy, a treatment whose role is still not fully established in this context [5,9]. Additionally, a reliable predictor of LNM can serve as a crucial selection criterion for future liver transplantation in patients with cholangiocarcinoma, given that LNM is a major contraindication to this procedure.

The present study aimed (i) to propose a novel approach for predicting LNM in ECC using CT imaging and (ii) to compare the predictive performance of the CT-based coefficient of variation (CV) value with that of the MRI ADC value and PET/CT SUV to demonstrate its superior accuracy in predicting LNM. Our approach is unique in that it uses the CV of CT values extracted from multiple lymph nodes in a single patient. This approach is hypothesized to provide a more robust and informative measure of lymph node heterogeneity than conventional metrics.

## Materials and methods

### Data sources and patients population

A retrospective analysis was conducted on 149 patients with ECC who underwent curative resection in our department from April 2012 to December 2024. The study protocol was approved by the Institutional Review Board of Tokyo Medical University (approval number: T2025-0094). The requirement for informed consent was waived owing to the retrospective nature of this study. All data accessed for this research purpose in November 20^th^, 2025.

Clinical and pathological data of all patients were collected and reviewed. The final diagnoses of both the disease itself and the presence of LNM were definitively determined via histopathological examination of surgically resected specimens.

### Assessment of imaging findings

**Fig 1:**
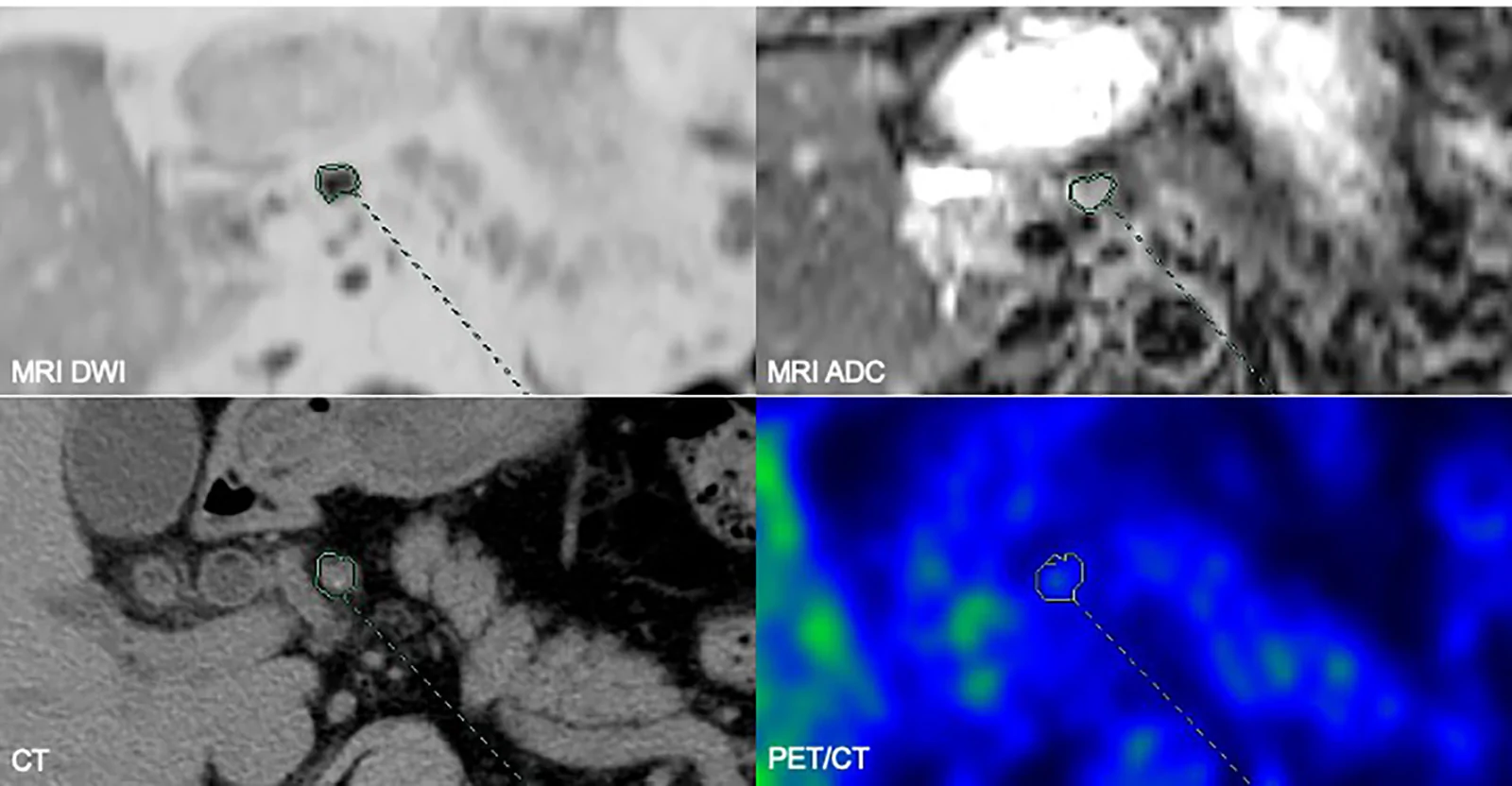
Workflow of multimodal imaging analysis for lymph node evaluation. Lymph nodes of interest were first identified on diffusion-weighted imaging (DWI), and the apparent diffusion coefficient (ADC) values were obtained from co-registered ADC maps. The same lymph nodes were then localized on contrast-enhanced CT, where morphometric (short- and long-axis diameters, aspect ratio) and densitometric parameters (mean CT value, coefficient of variation of CT values) were automatically calculated. For patients who underwent PET/CT, the same lymph nodes were analyzed on the corresponding slice, and the maximum standardized uptake value (SUV_max_) was measured as an indicator of metabolic activity. Multiple lymph nodes per patient were analyzed using this multimodal approach.

Two radiologists and two experienced surgeons blinded to the clinical data independently reviewed all CT, MRI, and PET/CT images. Any disagreement was resolved by consensus. In this study, we selected and analyzed the imaging data closest to the time of surgery for all patients. While CT and MRI scans were routinely performed preoperatively in all cases, PET-CT scans were not performed in 45 cases due to timing issues; therefore, the analysis was conducted using the remaining cases. For each patient, a comprehensive analysis of imaging parameters was performed (Fig 1). First, the lymph nodes of interest were identified using DWI sequences. Subsequently, the corresponding ADC value was measured by drawing a region of interest on the co-registered ADC map. Next, the same lymph node was identified in the corresponding slice of the contrast-enhanced CT image. Several morphometric and densitometric features, including short-axis diameter, long-axis diameter, aspect ratio, mean CT value (in Hounsfield units), and CV of CT values were automatically calculated by a dedicated software. Finally, for patients who underwent PET/CT, the SUV for the same lymph node was measured in the corresponding PET/CT slice. Specifically, the maximum SUV (SUV_max_) was used as a representative value to quantify metabolic activity. This process was performed using multiple lymph nodes extracted from each patient.

### Statistical analysis

All statistical analyses were performed using SPSS software version 29 (IBM Corp., Armonk, NY, USA). Continuous variables were analyzed using Student’s t-test, whereas categorical variables were examined using the chi-square test or Fisher’s exact test. The performance of the CV of CT values, ADC value of MRI, and SUV of PET/CT in predicting LNM was evaluated using receiver operating characteristic (ROC) curves. The area under the curve (AUC), sensitivity, specificity, and accuracy of each parameter were calculated. Statistical significance was set at a two-sided *p*-value of <0.05.

### Ethical consideration

This research has been approved by Tokyo Medical University Ethics Committee.

## Results

### Patients’ characteristics

A total of 149 patients with ECC who underwent curative resection were included in this study (Table 1). The mean age of the cohort was 73 years (range: 40–87 years). The study group comprised 97 males (65%) and 52 females (35%). With respect to pathological diagnosis, 67 patients (45%) had hilar cholangiocarcinoma, whereas 82 (55%) had common bile duct cancer. The mean tumor marker values were 3.0 ng/mL for CEA (range: 0.8–55.3) and 36.0 U/ mL for CA19−9 (range: 2.1–12000). The most common surgical procedures were pancreaticoduodenectomy (54%) and hepatectomy (34%). LNM was pathologically confirmed in 55 patients (37%), whereas 94 patients (63%) were LNM-negative. Most patients were classified as stage II (45%) according to the American Joint Committee on Cancer tumor staging system, with other stages distributed as follows: stage I (23%), stage III (20%), and stage IV (12%).

**Table 1:** Patients clinicopathological characteristics.

Characteristics	Total no. of patients = 149
Age (years)	Mean: 73, Range: 40 – 87
Sex	
Male	97 (65%)
Female	52 (35%)
Pathological diagnosis	
Hilar cholangiocarcinoma	67 (45%)
Common bile duct cancer	82 (55%)
Tumor marker	
CEA (ng/ml)	Mean: 3.0, Range: 0.8 – 55.3
CA19-9 (U/ml)	Mean: 36.0, Range: 2.1 – 12000
Surgical procedure	
Hepatectomy	51 (34%)
Pancreaticoduodenectomy	81 (54%)
HPD	14 (9%)
Others	3 (3%)
Lymph node metastasis	
N0	94 (63%)
N1	55 (37%)
AJCC tumor staging	
I	32 (23%)
II	67 (45%)
III	30 (20%)
IV	20 (12%)

CEA; Carcinoembryonic Antigen, CA19−9; Carbohydrate Antigen 19−9, HPD; Hepatopancreatoduodenectomy, AJCC; American Joint Committee on Cancer

### Comparison of clicopathological factors between lymph nodes with and without metastasis

Clinicopathological factors other than radiological findings between lymph node with and without metastasis were compared and summarized (Table 2). No significant differences were observed for any of the factors. It was difficult to predict lymph node metastasis based on clinical and pathological factors.

**Table 2:** Patients clinicopathological characteristics stratified by lymph node metastasis.

Characteristics	LNM negative	LNM positive	p-value
	(N=94)	(N=55)	
Age	Median: 73	Median: 73	0.756
	Range: 40-87	Range: 47-86	
Gender			0.223
Male	65 (69%)	32 (58%)	
Female	29 (31%)	23 (42%)	
Pathological diagnosis			0.349
Hilar cholangiocarcinoma	39 (41%)	28 (51%)	
Common bile duct cancer	55 (59%)	27 (49%)	
Tumor marker			
CEA (ng/ml)	Median: 3.0	Median: 3.2	0.961
	Range: 0.8-55.3	Range: 1.4-9.1	
CA19-9 (U/ml)	Median: 29.2	Median: 77.4	0.991
	Range: 2.1-12000	Range: 2.1-12000	
Surgical procedure			0.349
Hepatectomy	29 (31%)	22 (40%)	
Pancreaticoduodenectomy	54 (57%)	27 (49%)	
HPD	9 (10%)	5 (9%)	
Others	2 (2%)	1 (2%)	

CEA; Carcinoembryonic Antigen, CA19−9; Carbohydrate Antigen 19−9, HPD; Hepatopancreatoduodenectomy

### Comparison of radiological findings between lymph nodes with and without metastasis

Comparison of radiological findings between lymph nodes with and without metastasis revealed several significant factors (Table 3). On CT, short-axis diameter (*p* < 0.001), long-axis diameter (*p* < 0.001), aspect ratio (*p* = 0.025), and CV of CT values (*p* < 0.001) were identified as significant predictors of LNM. Furthermore, the mean ADC (*p* < 0.001) from MRI and the maximum SUV (SUV_max_) (*p* < 0.001) from FDG PET/CT were significant predictors of LNM.

**Table 3:** Radiological findings compared with negative and positive lymph node metastasis.

Valuables	LN meta (-) N=343	LN meta (+) N=81	p-value
CT findings			
Short axis (mm)	4.8 (2.4 – 10.1)	6.4 (3.7 – 11.8)	<0.001
Long axis (mm)	5.8 (3.2 – 14.7)	8.1 (4.5 – 17.4)	<0.001
Aspect ratio	0.87 (0.29- 0.98)	0.81 (0.49 – 1.00)	0.025
CT value average (HU)	23.5 (3.8 – 55.5)	23.5 (4.2 – 52.8)	0.957
CT value CV	0.26 (0.09 – 1.39)	0.46 (0.21 – 2.71)	<0.001
MRI findings			
Mean ADC value (x10^-3^mm^3^/s)	1.79 (1.24 – 2.83)	1.72 (1.21 – 2.25)	<0.001
FDG PET-CT findings			
SUV max value	1.85 (1.23 – 2.13)	1.97 (1.49 – 2.55)	<0.001

CV; coefficient of variation, ADC; apparent diffusion coefficient, SUV max; maximum standardized uptake value

### Predictive radiological findings for LNM

Both univariate and multivariate analyses were performed to identify independent radiological factors predictive of LNM (Table 4). Univariate analysis showed that the CT aspect ratio, CV of CT values, mean MRI ADC value, and FDG PET-CT SUV_max_ were significant predictors. Multivariate analysis revealed that the CV of CT values was the most powerful independent predictor of LNM. A CV of CT values exceeding 0.35 had an odds ratio (OR) of 11.723 (95% confidence interval [CI]: 4.613–29.793, *p* < 0.001). Other significant independent predictors were FDG PET/CT SUV_max_ (HR: 6.927, 95% CI: 2.879–16.669, *p* < 0.001) and CT aspect ratio (OR: 3.390, 95% CI: 1.412–8.130, *p* = 0.006). Notably, the mean MRI ADC value was significant in the univariate analysis, but was not an independent predictor in the multivariate analysis (*p* = 0.725).

**Table 4:** Predictive radiological findings for lymph node metastasis.

Valuables	Univariate analysis			Multivariate analysis		
	OR	95%CI	p-value	OR	95%CI	p-value
CT findings						
Aspect ratio						
>0.83	1			1		
<0.83	2.114	1.294 - 3.448	0.003	3.39	1.412 – 8.130	0.006
CT value CV						
<0.35	1	4.183 – 12.426	<0.001	1	4.613 – 29.793	<0.001
>0.35	7.209			11.723		
MRI findings						
Mean ADC value						
>1.75	1			1		
<1.75	2.676	1.629 – 4.395	<0.001	1.155	0.517 – 2.582	0.725
FDG PET-CT findings						
SUV max value						
<1.95						
>1.95	1	2.073 – 8.184	<0.001	1	2.879 – 16.669	<0.001
	4.119			6.927		

CV; coefficient of variation, ADC; apparent diffusion coefficient, SUV max; maximum standardized uptake value

### Diagnostic performance in Patients with ECC

**Fig 2:**
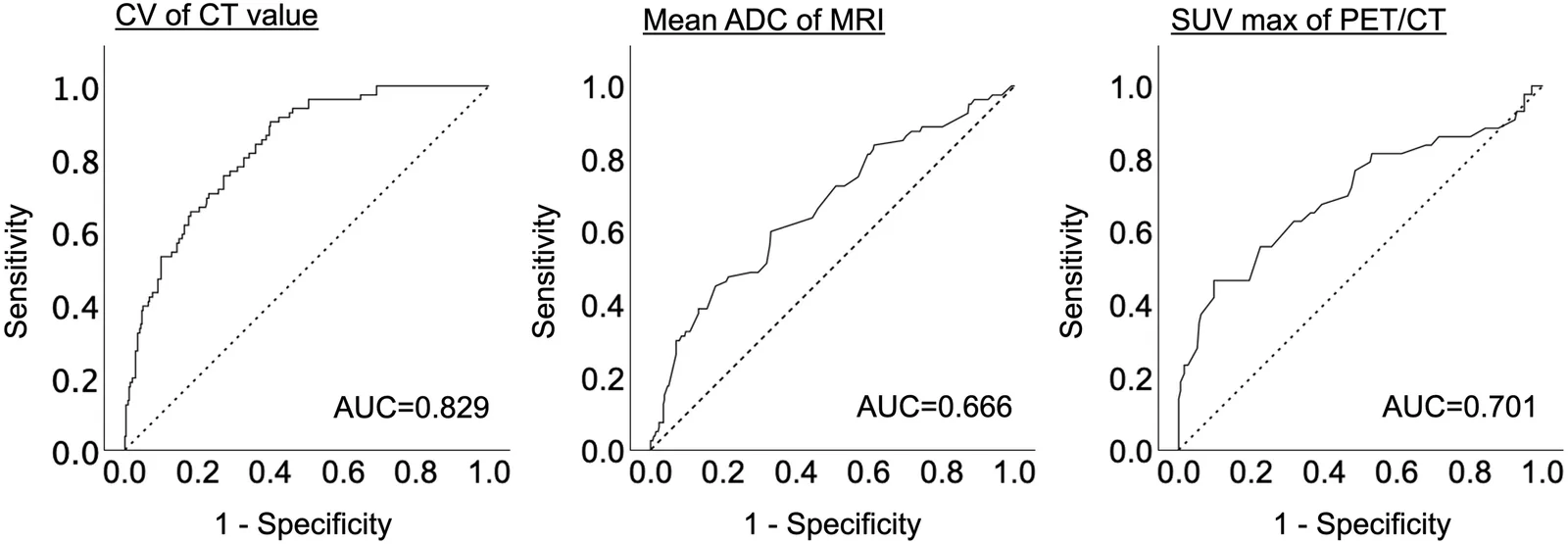
Comparison of ROC curves for CT coefficient of variation, MRI ADC, and PET SUV_max_ in lymph node prediction. The area under the curve (AUC) for the coefficient of variation (CV) of CT values was 0.829, outperforming the mean apparent diffusion coefficient (ADC) value from MRI (AUC = 0.666) and the maximum standardized uptake value (SUV_max_) from FDG PET/CT (AUC = 0.701).

Table 4 shows the diagnostic performance of the three radiological parameters in predicting LNM. The CV of CT values exhibited a sensitivity of 72.8%, specificity of 72.9%, and accuracy of 72.9%. The mean ADC value from MRI showed a sensitivity of 56.3%, specificity of 67.5%, and accuracy of 65.4%. The SUV_max_ from PET/CT achieved a sensitivity, specificity, and accuracy of 65.8%, 72.5%, and 70.8%, respectively. When the predictive value was assessed using ROC curve analysis, the AUC for the CV of CT values was 0.829, which was superior to that of the mean ADC value from MRI (0.666) and SUV_max_ from FDG PET-CT (0.701) (Fig 2).

Collectively, these results indicated that the CV of CT values was the most effective diagnostic method for predicting LNM among the three modalities. Its superior performance was demonstrated by its higher sensitivity, specificity, accuracy, and, most notably, the highest AUC value.

## Discussion

Accurate preoperative prediction of LNM is of paramount importance in determining the optimal treatment strategy for ECC. LNM is a crucial prognostic factor, and its presence often necessitates the consideration of neoadjuvant chemotherapy, as surgery alone may be insufficient [10–12]. Furthermore, while liver transplantation offers a potential curative option for cholangiocarcinoma, LNM remains a major contraindication. Thus, precise preoperative diagnosis of LNM allows for a more judicious selection of transplant candidates [13]. Therefore, our study aimed to validate the use of the CV of CT values as a novel method for predicting LNM in patients with ECC. Our findings demonstrate that the CV of CT values exhibits superior predictive performance compared with conventional MRI ADC and PET/CT SUV_max_. The most significant finding of this study was that CV of CT values was the most powerful independent predictor of LNM. Our approach of utilizing the CV of CT values for predicting LNM offers several notable advantages and unique aspects compared with existing diagnostic methods.

First, it has a high degree of originality. While the concept of quantifying heterogeneity or uniformity in imaging has been employed for the prognosis of other cancer types, the specific application of the CV of CT values for predicting LNM in biliary cancer is largely unreported. For instance, in non-small cell lung cancer (NSCLC), the CV of both lymph nodes and primary tumors on 18F‑FDG PET/CT images have been reported to be predictive factors for overall survival (OS) [14]. That study noted that a higher CV in the lymph nodes was associated with poor prognosis, while an inverse correlation was observed with the CV of the primary tumor. Similarly, in multiple myeloma, the CV of focal lesions (FL) on FDG-PET/CT was identified as an independent prognostic factor in the multivariate analysis [15]. Another retrospective study on NSCLC also suggested that the homogeneity (low CV) of FDG uptake in the lymph nodes was associated with a more favorable prognosis, again showing a trend opposite to that of the CV of the primary tumor [16]. Specifically, Hua et al. reported that “CV was significantly higher in metastatic lymph nodes than in healthy lymph nodes,” which supports the concept that CV reflects the heterogeneity of metastatic lesions. However, while these studies focused on PET/CT SUV, our study used the CV of Hounsfield units (HU) from plain CT images, specifically for LNM prediction, making our approach highly novel. It strongly suggests that the CV of CT values reflects internal tissue heterogeneity, which serves as the biological basis for predicting LNM. In general, a normal lymph node has a relatively uniform tissue structure, resulting in a narrow distribution of CT values and a low CV. When cancer cells infiltrate and proliferate within the lymph nodes, the normal lymphatic architecture is disrupted, creating a heterogeneous mix of high and low cell densities. Furthermore, as the tumor grows, irregular necrosis, fibrosis, or neoangiogenesis may occur, which broadens the distribution of CT values and consequently increases the CV. Thus, the CV of CT values is thought to reflect subtle internal structural changes characteristic of metastatic lesions that cannot be captured by macro-level information, such as lymph node size or mean CT value. This is considered the biological rationale for why the CV of CT values exhibits superior predictive ability compared with conventional simple indicators.

Second, our methodology is simple and clinically practical. Many recent studies have utilized complex radiomics models to predict LNM, which often requires specialized software and intricate algorithms [5,6]. Although these models can achieve a high predictive accuracy, their complexity poses a significant barrier to their implementation in routine clinical practice. In contrast, the CV of CT values can be easily calculated using standard imaging analysis tools without the need for sophisticated equipment or complex processing, making them highly accessible for widespread clinical applications.

Third, the CV of CT values demonstrated superior predictive performance over conventional imaging markers. Our ROC curve analysis yielded an AUC of 0.829 for the CV of CT values, which surpassed those of the MRI ADC value (0.666) and the PET/CT SUV_max_ (0.701). While previous studies have highlighted the usefulness of DWI and PET/CT, their diagnostic accuracy is limited [3,4]. Our results suggest that the CV of CT values, as a quantitative measure of internal heterogeneity, may be more effective than traditional functional imaging metrics in accurately identifying LNMs.

Finally, by analyzing multiple lymph nodes from each patient, our study provides a more comprehensive and objective evaluation of the overall lymph node status, thereby reducing potential bias compared to analyses that focus on a single representative lymph node.

Our study had several limitations. First, because this was a retrospective, single-institution study, the generalizability of our findings is limited. Prospective, multi-institutional studies with larger patient cohorts are required to validate our results. Second, the relatively small sample size may have affected the robustness of the statistical analysis, particularly multivariate analysis. Furthermore, we must acknowledge that some advanced radiomics models have reported even higher AUC values (exceeding 0.90) than our CV of CT values [17,18]. However, the simplicity and high clinical feasibility of our method provide a significant advantage over more complex approaches. Finally, the precise pathological correlation between the CV CT values remains unclear. Future studies should aim to correlate this metric with detailed histopathological findings to better understand its biological basis.

In conclusion, the CV of CT value represents a novel and potent tool for the preoperative diagnosis of LNM. Its accurate prediction of LNM could become an essential factor in clinical decision-making, influencing the selection of patients for neoadjuvant chemotherapy and potentially informing the criteria for liver transplantation in the future.

## Conclusion

Our study demonstrated that the CV of CT values is a promising and highly effective parameter for preoperatively predicting LNM in ECC. This novel approach offers superior diagnostic performance over conventional MRI ADC and PET/CT SUV_max_. Given its simplicity and accessibility, the CV of CT values represents a valuable addition to preoperative diagnostic findings, potentially improving patient selection for neoadjuvant therapies and surgical strategies.

## Supporting information

S1 Data LNmeta ECC raw data. (XLSX)

## References

[1] Liao P, Cao L, Chen H, Pang S-Z. Analysis of metastasis and survival between extrahepatic and intrahepatic cholangiocarcinoma: A large population-based study. Medicine (Baltimore). 2021;100(16):e25635. doi: 10.1097/MD.0000000000025635 33879742 PMC8078350

[2] Khomiak A, Ghaffar SA, Rodriguez Franco S, Ziogas IA, Cumbler E, Gleisner AL, et al. Prognostic Significance of Lymph Node Ratio in Intrahepatic and Extrahepatic Cholangiocarcinomas. Cancers (Basel). 2025;17(2):220. doi: 10.3390/cancers17020220 39858002 PMC11764393

[3] Hosokawa I, Hayano K, Furukawa K, Takayashiki T, Kuboki S, Takano S, et al. Preoperative Diagnosis of Lymph Node Metastasis of Perihilar Cholangiocarcinoma Using Diffusion-Weighted Magnetic Resonance Imaging. Ann Surg Oncol. 2022;29(9):5502–10. doi: 10.1245/s10434-022-11931-4 35639292

[4] Yang C, Huang M, Li S, Chen J, Yang Y, Qin N, et al. Radiomics model of magnetic resonance imaging for predicting pathological grading and lymph node metastases of extrahepatic cholangiocarcinoma. Cancer Lett. 2020;470:1–7. doi: 10.1016/j.canlet.2019.11.036 31809800

[5] Chen P, Yang Z, Ning P, Yuan H, Qi Z, Li Q, et al. To accurately predict lymph node metastasis in patients with mass-forming intrahepatic cholangiocarcinoma by using CT radiomics features of tumor habitat subregions. Cancer Imaging. 2025;25(1):19. doi: 10.1186/s40644-025-00842-8 40011960 PMC11863903

[6] Zhang S, Huang S, He W, Wei J, Huo L, Jia N, et al. Radiomics-Based Preoperative Prediction of Lymph Node Metastasis in Intrahepatic Cholangiocarcinoma Using Contrast-Enhanced Computed Tomography. Ann Surg Oncol. 2022;29(11):6786–99. doi: 10.1245/s10434-022-12028-8 35789309

[7] Ruys AT, Bennink RJ, van Westreenen HL, Engelbrecht MR, Busch OR, Gouma DJ, et al. FDG-positron emission tomography/computed tomography and standardized uptake value in the primary diagnosis and staging of hilar cholangiocarcinoma. HPB (Oxford). 2011;13(4):256–62. doi: 10.1111/j.1477-2574.2010.00280.x 21418131 PMC3081626

[8] Kim NH, Lee SR, Kim YH, Kim HJ. Diagnostic Performance and Prognostic Relevance of FDG Positron Emission Tomography/Computed Tomography for Patients with Extrahepatic Cholangiocarcinoma. Korean J Radiol. 2020;21(12):1355–66. doi: 10.3348/kjr.2019.0891 32767862 PMC7689144

[9] Wang Y, Shao J, Wang P, Chen L, Ying M, Chai S, et al. Deep Learning Radiomics to Predict Regional Lymph Node Staging for Hilar Cholangiocarcinoma. Front Oncol. 2021;11:721460. doi: 10.3389/fonc.2021.721460 34765542 PMC8576333

[10] Choi SB, Park SW, Kim KS, Choi JS, Lee WJ. The survival outcome and prognostic factors for middle and distal bile duct cancer following surgical resection. J Surg Oncol. 2009;99(6):335–42. doi: 10.1002/jso.21238 19226533

[11] Kitagawa Y, Nagino M, Kamiya J, Uesaka K, Sano T, Yamamoto H, et al. Lymph node metastasis from hilar cholangiocarcinoma: audit of 110 patients who underwent regional and paraaortic node dissection. Ann Surg. 2001;233(3):385–92. doi: 10.1097/00000658-200103000-00013 11224627 PMC1421255

[12] Kiriyama M, Ebata T, Aoba T, Kaneoka Y, Arai T, Shimizu Y, et al. Prognostic impact of lymph node metastasis in distal cholangiocarcinoma. Br J Surg. 2015;102(4):399–406. doi: 10.1002/bjs.9752 25611179

[13] Sapisochín G, Fernández de Sevilla E, Echeverri J, Charco R. Liver transplantation for cholangiocarcinoma: Current status and new insights. World J Hepatol. 2015;7(22):2396–403. doi: 10.4254/wjh.v7.i22.2396 26464755 PMC4598610

[14] Kim D-H, Jung J-H, Son SH, Kim C-Y, Hong CM, Oh J-R, et al. Prognostic Significance of Intratumoral Metabolic Heterogeneity on 18F-FDG PET/CT in Pathological N0 Non-Small Cell Lung Cancer. Clin Nucl Med. 2015;40(9):708–14. doi: 10.1097/RLU.0000000000000867 26098287

[15] Pellegrino S, Origlia D, Di Donna E, Lamagna M, Della Pepa R, Pane F, et al. Coefficient of variation and texture analysis of 18F-FDG PET/CT images for the prediction of outcome in patients with multiple myeloma. Ann Hematol. 2024;103(9):3713–21. doi: 10.1007/s00277-024-05905-7 39046513 PMC11358233

[16] Hua J, Li L, Liu L, Liu Q, Liu Y, Chen X. The diagnostic value of metabolic, morphological and heterogeneous parameters of 18F-FDG PET/CT in mediastinal lymph node metastasis of non-small cell lung cancer. Nucl Med Commun. 2021;42(11):1247–53. doi: 10.1097/MNM.0000000000001456 34269750

[17] Zhan P-C, Yang T, Zhang Y, Liu K-Y, Li Z, Zhang Y-Y, et al. Radiomics using CT images for preoperative prediction of lymph node metastasis in perihilar cholangiocarcinoma: a multi-centric study. Eur Radiol. 2024;34(2):1280–91. doi: 10.1007/s00330-023-10108-1 37589900

[18] Liang X, Cai W, Liu X, Jin M, Ruan L, Yan S. A radiomics model that predicts lymph node status in pancreatic cancer to guide clinical decision making: A retrospective study. J Cancer. 2021;12(20):6050–7. doi: 10.7150/jca.61101 34539878 PMC8425217

